# Effect of Different Acid and Base Potassium Ferrate Pretreatment on Organic Acid Recovery by Anaerobic Digestion of Sludge

**DOI:** 10.3390/ijerph192215093

**Published:** 2022-11-16

**Authors:** Mengjia Tian, Feng Liu, Jiawen Guo, Wei Li, Mao Zhang, Xiang Li

**Affiliations:** 1 School of Environmental Science and Engineering, Suzhou University of Science and Technology, Suzhou 215009, China; 2 Jiangsu Collaborative Innovation Center of Technology and Material of Water Treatment, Suzhou University of Science and Technology, Suzhou 215009, China; 3 State Key Laboratory of Urban Water Resource and Environment, Harbin Institute of Technology, Harbin 150090, China

**Keywords:** potassium ferrate, sludge reduction, anaerobic digestion, VFAs

## Abstract

Potassium ferrate has strong oxidation in both acid and alkali environments, which has attracted extensive attention. However, the impact of the pH environment on this coupling process with the goal of resource recovery has not received attention. Under the goal of the efficient recovery of organic acid, the changes of solid–liquid characteristics of sludge after acid and alkaline ferrate pretreatment and during anaerobic digestion were discussed. The results showed that compared with blank control groups, after alkaline ferrate pretreatment, the volatile suspended solids (VSSs) decreased the most, reaching 28.19%. After being pretreated with alkaline ferrate, the sludge showed the maximum VFA accumulation (408.21 COD/g VSS) on the third day of digestion, which was 1.34 times higher than that of the acid ferrate pretreatment. Especially in an alkaline environment, there is no need to add additional alkaline substances to adjust the pH value, and the effect of sludge reduction and acid production is the best.

## 1. Introduction

According to statistics, in 2019, the annual wet sludge production in China was as high as 5.95 million tons. If all urban sewage is treated, the sludge production will exceed 8.4 million tons, accounting for approximately 3% of the total solid waste in China [1]. Excess sludge contains heavy metals and refractory pollutants, and its pathogens and parasites may infect human beings and threaten human health [2]. Therefore, it is an urgent issue to address. However, excess sludge is rich in organic matter, which is a potential raw material for producing energy and high-value-added chemicals and can be recycled [3]. Therefore, it is not appropriate to simply classify excess sludge as “hazardous waste” that needs urgent disposal, and further research should be made regarding the utilization of sludge resources [4].

At present, there are many methods of excess sludge treatment and disposal such as sanitary landfills, sludge incineration, hydrothermal hydrolysis [5], and so on, but anaerobic digestion is the simplest and most important method in the excess sludge treatment process. It can not only stabilize and reduce excess sludge but also convert degradable bio-organic components into renewable resources such as methane and hydrogen [6]. The current study found that microorganisms such as denitrifying bacteria and phosphorus-accumulating organisms preferentially take acetic acid and propionic acid in volatile fatty acids (VFAs) as carbon sources. Furthermore, when VFAs are used as a carbon source, the nitrogen and phosphorus removal efficiency is higher than other carbon sources such as methanol and ethanol [7]. Therefore, studying the effective recovery of VFAs can not only alleviate the problem of insufficient carbon sources in sewage treatment plants but also help to achieve high-efficiency nitrogen and phosphorus removal [8]. In addition, VFAs have other high economic benefits, such as being used as raw material for producing poly-hydroxyalkanoate (PHA) [9,10] or realizing bioelectric energy production through bio-electrochemical systems [11]. Therefore, developing a method of improving the amount of VFAs in sludge digestion and resource recovery has become a hot topic. Different pretreatment methods have different effects on the recovery of VFAs from sludge. Yan et al. [12] using ultrasound pretreatment and digestion, VFA accumulation reached 3109.8 mg chemical oxygen demand (COD)/L, which was more than twice that of the blank. Xin et al. [13] tried to pretreat sludge with a CaO agent, and when the dosage of CaO was 0.07 g/g total suspended solid (TSSs), the accumulation of VFAs was 327.8 mg COD/g volatile suspended solids (VSSs), which was nearly 1.5-fold higher than the blank. Xu et al. [14] found that when 62.6 mg free ammonia (FA)/L pretreatment was combined with 0.04 g rhamnolipid/g TSS, the maximum yield of VFAs was 324.7 mg COD/g VSS, which was 5.95 times that of the blank group. 

Potassium ferrate, as a green strong oxidant, can not only destroy the aggregate structure of sludge but also produce Fe^3+^ to help flocculation and sedimentation and react with PO_4_^3−^-P to form the Fe-P precipitate so as to achieve the effect of removing PO_4_^3−^-P [15,16]. Therefore, potassium ferrate has attracted much attention in the process of sludge pretreatment. For example, Li et al. [17] showed that under a ferrate dosage of 500 mg Fe/g VSS, the maximum VFAs reached 322.6 mg COD/g VSS on the fifth day of digestion, which was 2.39 times that of the blank peak value. On the basis of Li et al. [17], He et al. [15] suggested that potassium ferrate be used for pretreatment and then fresh sludge was added for digestion. When the ferrate dosage was 56 mg Fe/g TSS, the maximum output of VFAs reached 343 mg COD/g VSS within 5 days, which was 6.72 times that of the blank control group. Through comparison with anaerobic digestion, it was found that the effect of adding fresh sludge to the ferrate-pretreated sludge to produce VFAs is better than that of direct anaerobic digestion after ferrate pretreatment by Li et al. [17]. It is speculated that the strong oxidation performance of potassium ferrate can not only fully oxidize the macromolecular sludge aggregates but also destroy the microbial cell structure. So, once the potassium ferrate concentration is too high, it will slow down the anaerobic digestion reaction rate. In addition, potassium ferrate is expensive, and if the appropriate dosage of potassium ferrate can be found, this will not only directly save on the economic cost but also maintain its microbial activity. Based on the above problems, Li et al. [16] found that when the dosage of potassium ferrate was 20 mg Fe/g suspended solids (SSs), it could reach the oxidation limit without destroying the microbial cell structure, and the dosage of potassium ferrate was less than half of that of Li et al. [17] or He et al. [15]. However, Li et al. [16] focused on the reduction effect of basic ferrate on sludge but lacked research on the recovery of small molecular organic acids. In addition, potassium ferrate has strong oxidation properties in both acid and base environments. At present, the research on potassium ferrate oxidation sludge is mostly carried out under alkaline conditions [18]. Therefore, the effect of ferrate on sludge pretreatment and anaerobic digestion recovery of organic matter under different acid and base environments needs further study.

Therefore, based on the results of Li et al. [16], sludge was pretreated with a low dose of potassium ferrate and then digested. On the premise that ferrate destroys cell aggregation without destroying microbial activity, we measured the changes of solid–liquid characteristics of sludge and deeply analyzed the changes in the microbial community after acid and alkaline ferrate pretreatment and during anaerobic digestion to explore the effects of acidic and basic ferrate on sludge oxidation pretreatment and anaerobic digestion. 

## 2. Materials and Methods

### 2.1. Characteristics of Excess Sludge

The experimental sludge was taken from the excess sludge of the A^2^O process in the final sedimentation tank of a sewage treatment plant in Suzhou. The wastewater treatment capacity of this plant is 80,000 t/d. The sludge return ratio (R) of the A^2^O process is 80%, the mixed sludge concentration (X) of the process is 150%, and the sludge retention time (SRT) of the process is 25 d. After simple sieving, washing, and settling, the sludge was stored in a refrigerator at 4 °C. The median particle diameter (Dx (50)) of raw sludge is 1749.37 μm, the volume ratio of sludge after standing for 30 min (in percentage, %) (SV_30_ ratio) is 48.00%, and other basic properties are shown in Table 1.

### 2.2. Experimental Method

The sludge was divided into 5 parts (labeled as S1, S2, S3, S4, and S5) and each sample was 1000 mL. Among them, S1, S2, and S3 were used as control groups, which were a blank, an acidic blank, and an alkaline blank, respectively. S1 maintains the raw sludge pH and then a 4 mol/L KOH solution and a 12 mol/L HCl solution were used to adjust the pH of the remaining beakers (2 L) to 3, 11, 3, and 11 in turn. Then, the 5 beakers were placed in the blender (ZR4-6, ZhongRun) and stirred at a speed of 120 r/min. During the stirring process, 35 mL of potassium ferrate at a concentration of 20,000 mg/L was rapidly added to S4 and S5. After 2 h, 200 mL of the mixed sludge was retained from each beaker for analysis.

After pretreatment, except for the alkaline blank and alkaline potassium ferrate components, the pH of the remaining 3 groups of sludge was adjusted to 7 [19]. All 5 samples were transferred to 1 L serum bottles and the subsequent anaerobic digestion experiment was carried out in a constant temperature shaker (THZ-320, JingHong, China) at 35 °C and a rotational speed of 120 r/min. During this period, the supernatant of each group was taken every 24 h to measure the change in sludge supernatant substance concentrations for 5 consecutive days (the samples were labeled S1′, S2′, S3′, S4′, and S5′ after 5 days of digestion). Two parallel experiments were conducted for each group.

### 2.3. Determination Index and Analysis Method

SS, VSS, the sludge volume index (SVI), the sludge settling velocity (SV_30_), and pH (PHS-3E, LeiCi) were determined by standard methods [20]. NH_4_^+^-N, total nitrogen (TN), PO_4_^3−^-P, and total phosphorous (TP) were determined by an Ultraviolet-visible Spectrophotometer (Uvmini-1280, SHIMADZU, China) [20]. COD was determined by the hash rapid digestion method (HACH, DRB 200) [20]. Dx (50) was measured by a laser particle size analyzer (MASTERSIZER 3000, Malvern, Britain). 

### 2.4. VFAs Analysis Method

At first, supernatants were filtered with 0.45 μm cellulose nitrate membrane filters, and then VFAs of all samples were determined by gas chromatography (GC8860, Agilent, America) [8]. A flame ionization detector (FID) was used to analyze the VFAs in the samples and Nitrogen (N_2_) as a carrier gas (velocity: 2 mL/min). A polarity column was chosen (HP-FFAP, 30 m × 0.32 mm × 0.25 μm), of which the injection volume was 0.5 μL and the split ratio was 15:1.

The initial temperatures of the column box, inlet, and detector were 85 °C, 250 °C, and 260 °C, respectively. We selected the temperature-programmed method (6 °C/min), which was stable for 3 min before rising to 175 °C. The detection process was 18 min in duration. 

### 2.5. Extracellular Polymeric Substances (EPS), PN, and PS Analysis Method

Firstly, the sludge mixture was centrifuged at different rotating speeds to be layered. We centrifuged 50 mL of the sludge mixture at 2000× *g*/min for 15 min (Slime EPS (S-EPS)); centrifuged 50 mL of the sludge mixture at 5000× *g*/min for 15 min (Loosely Bound EPS (L-EPS)); pretreated the sludge mixture via ultrasound (20 kHz, 480 W, 10 min); and then centrifuged it at 13,000× *g*/min for 15 min (Tightly Bound EPS (T-EPS)). Secondly, we filtered the supernatant with a 0.45 μm membrane for determination. 

The protein (PN) concentration was determined by Lowry–Folin spectrophotometry [11]. We added 2 mL of the BCA (bicinchonininc acid) reagent to a 0.1 mL sample, mixed it well, soaked it in a thermostat water bath (HSWS-420, Labtrip) for 30 min at 37 °C, and then measured the absorbance at 562 nm. The concentration of polysaccharide (PS) was determined by phenol-sulfuric acid spectrophotometry [12,13]. We diluted the samples to 2 mL, added 1 mL of 6% phenol and 5 mL of sulfuric acid, left the sample for 20 min, and then measured the absorbance at 490 nm. 

### 2.6. Microbiological Analysis

Microbial high-throughput sequencing was used to measure microorganisms [14]. Using the universal primer of the bacterial 16S V3-V5 region, the sludge samples before and after digestion were amplified 3 times and the amplified products of the same sample were mixed. The front primer 515F (5-GTGCCAGCMGCCGCGG-3) and the back primer 907R (5-CCGTCAATTCMT TTRAGTTT-3) were used. Polymerase Chain Reaction (PCR) products were purified by gel cutting, quantified by Qubit, equimolar mixed, used to build the sequencing library, and sequenced by illumina hiseq.

## 3. Results

### 3.1. Changes in Sludge Characteristics after Pretreatment

#### 3.1.1. Changes in Liquid Phase in Sludge after Pretreatment

By analyzing the concentrations of NH_4_^+^-N, TN, PO_4_^3−^-P, TP, COD, and VFAs, the change in the material release of the liquid phase in sludge before and after acid and base potassium ferrate pretreatment was analyzed (Table 2). 

After pretreatment, the COD of the blank control group (S1) in the liquid phase was 62 mg/L and the acid blank control group (S2) remained unchanged (71 mg/L), while the COD of the alkaline blank control group (S3) increased sharply (500 mg/L). After ferrate pretreatment, the COD increased dramatically, among which the concentration after acid ferrate treatment (S4) was 23.38 times higher than that of the blank control group (S1) and that after alkaline ferrate treatment (S5) was 43.55 times higher than that of the blank control group (S1). Li et al. [16] found that potassium ferrate showed high COD-releasing ability under acid and base environments, which was consistent with these experimental results. 

After pretreatment, the TN in the blank control group (S1) was 21 mg/L and the acid blank control group (S2) did not change much. TN was increased slightly in the alkaline blank control group (S3), which may be due to the fact that alkaline pretreatment also cracked the sludge [21]. The TN values after acid and alkaline ferrate pretreatments (S4, S5) both increased to 61 mg/L and 82 mg/L, respectively. This further indicated that under the action of ferrate, a large amount of macromolecular organic matter in sludge is oxidized and cracked and released into the liquid phase, and more organic matter in sludge is oxidized by ferrate to become soluble matter, which is beneficial for the subsequent hydrolysis [22]. After pretreatment, the NH_4_^+^-N in the blank control group (S1) was only 2 mg/L, and the NH_4_^+^-N in the acid and base blank control groups (S2, S3) did not change much, but after ferrate pretreatment (S4, S5), NH_4_^+^-N increased, especially after alkaline ferrate pretreatment (S5). There are two reasons for the increase in the NH_4_^+^-N concentration: On the one hand, ferrate oxidizes and cracks sludge, which releases intracellular substances; on the other hand, the reaction rate of ferrate and organic nitrogen in water is faster than inorganic nitrogen [23]. The NH_4_^+^-N in each group accounted for approximately 25% of TN, which indicated that most nitrogen existed in the liquid phase in the form of or”anic’nitrogen. 

After pretreatment, the PO_4_^3−^-P in the blank control group (S1) was 12 mg/L, and the PO_4_^3−^-P in the acid blank control group (S2) did not change much, while that in the alkaline blank control group (S3) increased a great deal, and the reason was the same as TN. After pretreatment with ferrate, the concentration of PO_4_^3−^-P increased. At this time, ferrate only had oxidation and flocculation effects and it did not form a Fe-P precipitate with phosphorus [16]. At the same time, the PO_4_^3−^-P concentration of S5 was higher than that of S4, and the sludge cracking effect was better, which was consistent with the above conclusions. The change in the TP concentration in the liquid phase was similar to that of PO_4_^3−^-P. However, unlike nitrogen, more than 75% of phosphorus existed in the form of PO_4_^3−^-P and only a small part was organic phosphorus. 

There was little difference in the concentration of VFAs in the five groups, which were 6.47 mg COD/g VSS, 16.8 mg COD/g VSS, 17.62 mg COD/g VSS, 22.08 mg COD/g VSS, and 26.38 mg COD/g VSS, respectively. This is because, at this time, the large organic particles in the solution are only oxidized into small organic particles and not into small molecular fatty acids.

**Figure 1:**
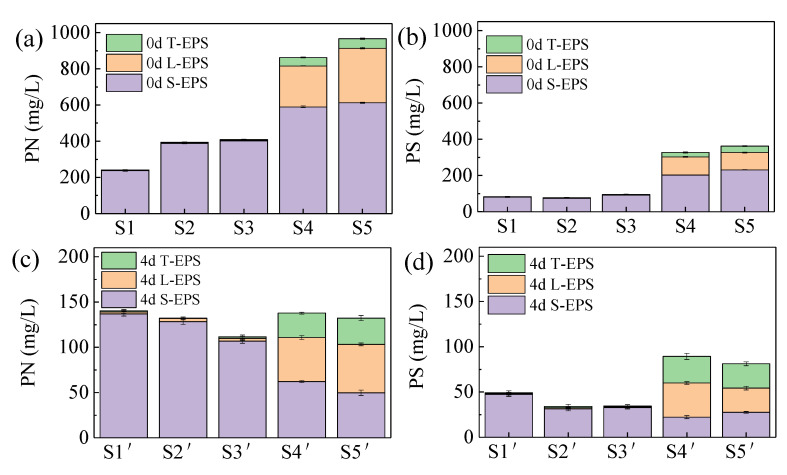
Changes in EPS in the liquid phase after ferrate pretreatment and digestion ((**a**) PN concentration after pretreatment, (**b**) PS concentration after pretreatment, (**c**) PN concentration after digestion, and (**d**) PS concentration after digestion).

Most organic matter exists in excess sludge or is entangled in the EPS matrix, so the presence of EPS hinders the hydrolysis of organic matter and slows down the sludge digestion rate [24]. At the same time, PN and PS are the key substances for sludge fermentation to produce acid, hydrolytic enzymes decompose PN and PS into amino acids and monosaccharides, and acidifying bacteria use amino acids and monosaccharides to produce VFAs [25]. After pretreatment, the composition and concentration of EPS in each group were different (Figure 1a,b). After pretreatment, the EPS of four groups (S2, S3, S4, and S5) increased from 325.13 mg/L (S1) to 471.37 mg/L, 504.15 mg/L, 1190.225 mg/L, and 1329.445 mg/L, respectively. This may be because ferrate destroyed the structure of sludge, which separated EPS and released it into the liquid phase. EPS increased slightly for the acid blank control group (S2) and the alkaline blank control group (S3), while EPS visibly increased after acid ferrate pretreatment (S4) and alkaline ferrate pretreatment (S5). This is mainly due to the release of S-EPS (S2 increased from 318.19 mg/L of S1 to 461.95 mg/L, S3 increased from 318.19 mg/L of S1 to 495.31 mg/L, S4 increased from 461.95 mg/L of S2 to 792.54 mg/L, and S5 increased from 495.31 mg/L of S3 to 843.515 mg/L). S-EPS surrounds the outermost layer of sludge flocs, while tightly bound EPS (TB-EPS), as the skeleton of the sludge polymer, is in the innermost layer of sludge [26]. Ferrate pretreatment mainly destroyed S-EPS but had little effect on TB-EPS, which further indicated that when the dosage of potassium ferrate was 20 mg Fe/g SS, ferrate only destroyed the external structure of cells during pretreatment but had little effect on the internal cell structure of microorganisms. PS and PN of three blank control groups did not change much, but after pretreatment with ferrate (S4, S5), they were greatly increased. After acid ferrate pretreatment (S4), compared with the acid blank control group (S2), PS and PN were increased by 320.9% and 119.2%, respectively. Compared with the alkaline blank control group (S3), PS and PN values after alkaline ferrate pretreatment (S5) were increased by 281.8% and 136.3%, respectively. At the same time, it was found that in all groups, the concentration of PN was obviously higher than that of PS, which was similar to Rong et al.’s findings [27].

#### 3.1.2. Changes of Solid Phase of Sludge after Pretreatment 

After pretreatment, the concentration and settling ability of sludge changed (Table 3). VSS of raw sludge was 6.1 g/L, and the acid blank control group (S2) and the alkaline blank control group (S3) did not change much. After acid and alkaline ferrate pretreatment (S4, S5), VSS decreased by 17.7% and 28.19%, respectively. Li et al. [16] found that the effect of potassium ferrate on sludge reduction was limited. When the dosage of potassium ferrate increased from 0 mg Fe/g SS to 20 mg Fe/g SS, the VSS of sludge obviously decreased, and when the dosage of ferrate increased from 20 mg Fe/g SS to 40 mg Fe/g SS, VSS only decreased by 0.08 g/L. After pretreatment, the SV_30_ ratio of the blank control group was 51%, the SV_30_ ratio of acid ferrate pretreatment (S4) decreased by 33.33%, and that of alkaline ferrate pretreatment (S5) decreased by 43.13%. The above two sets of data showed that the cracked sludge solid organic matter is dissolved into the liquid phase, and the destructive effect of alkaline ferrate is stronger than that of acid ferrate. Compared with the blank control group (S1), the SVI after pretreatment with ferrate (S4, S5) showed a downward trend and the treatability of subsequent sludge was improved. On the one hand, the decrease in SVI may be due to the direct reduction of the sludge volume via the conversion of the organic solid phase to the liquid phase, and on the other hand, it may be due to the flocculation of iron that made sludge gather. Li et al. [16] found that SVI remained basically unchanged after pretreatment with ferrate under the conditions of a sludge concentration of 11,187 mg/L, which may be related to the different initial sludge concentrations. 

Dx (50) changed greatly before and after pretreatment. The Dx (50) of the blank control groups (S1, S2, and S3) were dozens of times higher than that after pretreatment with ferrate (S4 and S5), and the Dx (50) of the sludge after alkaline ferrate pretreatment (S5) was 46.87 μm less than 69.03 μm after acid ferrate pretreatment (S4). Li et al. [2] pretreated the sludge with excessive ferrate, and the dosage of the agent was 0.9 g ferrate/g VSS. It was found that the Dx (50) of the sludge was 44.23 μm, and there was no significant difference in the degree of sludge cracking. At the same time, Zhang [28] found that the Dx (50) of sludge first decreased and then increased with the increase in the ferrate dosage from 0 to 1200 mg/L, and this was due to the re-aggregation of sludge particles under the flocculation of Fe^3+^ when the dosage of ferrate is high. From this, it can be seen that ferrate can directly break the sludge floc particles, but the appropriate concentration of potassium ferrate is the best choice. At the same time, it has a better cracking effect in an alkaline environment, which may be because ferrate is more stable in an alkaline environment [29].

### 3.2. Analysis of Pretreated Sludge Characteristics during Digestion

#### 3.2.1. Liquid Phase Analysis of Pretreated Sludge during Digestion

**Figure 2:**
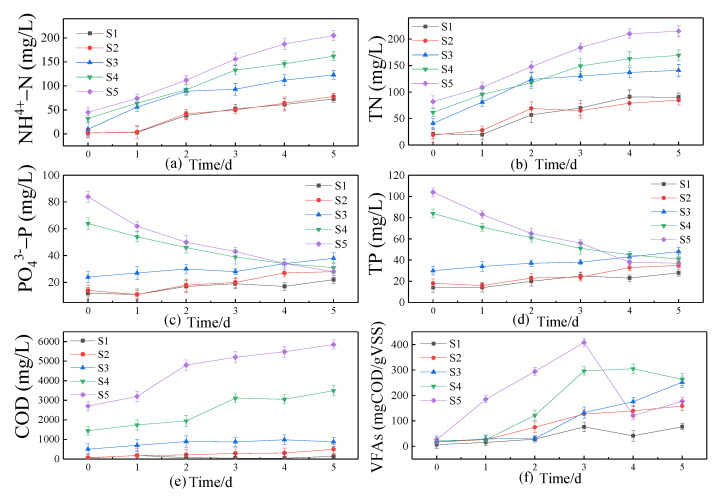
Variation of liquid pollutant concentration with digestion time ((**a**), (**b**), (**c**), (**d**), (**e**) and (**f**), respectively, are the changes of NH_4_^+^-N, TN, PO_4_^3−^-P, TP, COD and VFAs concentrations during 5 days).

The liquid release rate of NH_4_^+^-N, TN, COD, and other substances directly reflects the anaerobic digestion rate. The changes in pollutants in sludge digestion were different after different pretreatments (Figure 2). 

The COD of each group continued to rise within 5 days, which meant that the hydrolysis process happened rapidly. After 5 days of digestion, the COD of three blank control groups (S1, S2, and S3) were 139 mg/L, 493 mg/L, and 880 mg/L, respectively. After alkaline ferrate pretreatment (S5), the COD value (5850 mg/L) was higher than after acid ferrate pretreatment (S4) (3490 mg/L), and the COD release rate was 40.9 times, 7.5 times, 8.3 times, and 1.5 times that of S1, S2, S3, and S4, respectively. 

With the progress of the digestion reaction, the concentration of NH_4_^+^-N increased continuously. There are two main reasons for the increase in the NH_4_^+^-N concentration. On the one hand, intracellular organic matter is released after sludge oxidative cracking [22]. On the other hand, macromolecular organic matter releases NH_4_^+^-N during hydrolysis [30]. After 5 days of digestion, the NH_4_^+^-N concentration increased from 2 mg/L, 2 mg/L, 10 mg/L, 32 mg/L, and 45 mg/L to 73 mg/L, 78 mg/L, 123 mg/L, 162 mg/L, and 205 mg/L, respectively. It was found that NH_4_^+^-N can inhibit methanogenic bacteria from producing methane, which is beneficial to the recovery of small molecular organic acids [31]. The concentration of NH_4_^+^-N after pretreatment with ferrate (S4, S5) was higher, which indicated that they were more beneficial to acid production. Feng et al. [32] found that the addition of iron can improve the digestion rate, which is consistent with the above results. Furthermore, the percentage of NH_4_^+^-N to TN in five groups increased from 11.68%, 12.94%, 40.01%, 52.07%, and 55.24% to 81.46%, 92.65%, 87.49%, 95.62%, and 95.39%, respectively. In this experiment, after ferrate treatment (S4, S5), the proportion of organic nitrogen during digestion was the smallest and the proportion of NH_4_^+^-N was the largest, and these results were consistent with those of Li et al. [2]. The decrease in organic nitrogen may be due to the fact that the ferrate component rapidly oxidizes the pretreated small molecular organic matter into inorganic matter during digestion. At the same time, iron in the liquid phase provides trace elements for anaerobic microorganisms, which strengthens the activity of microorganisms [33].

During digestion, the concentration of PO_4_^3−^-P in three blank groups increased continuously. This is due to a great deal of PO_4_^3−^-P being released by phosphorus accumulation organisms (PAO) under anaerobic conditions [34]. The PO_4_^3−^-P release rate of the alkaline blank control group (S3) was faster than that of the blank control group (S1) and the acid blank control group (S2), which is because alkaline pretreatment (S3) accelerated the release of organic matter and anaerobic bacteria [21]. Under anaerobic conditions, PAO released a large amount of PO_4_^3−^-P, but after pretreatment with ferrate the concentration of PO_4_^3−^-P decreased. The main reason is that iron produced by ferrate oxidation plays a role in phosphorus removal, which causes PO_4_^3−^-P to migrate to the solid phase and form the Fe-P precipitate [35]. Furthermore, the formation rate of the Fe-P precipitate is faster than the release rate of liquid PO_4_^3−^-P, achieving the effect of phosphorus removal. In addition, on the premise of adding the same concentration of iron, on the fifth day of anaerobic digestion, after pretreatment with ferrate (S4, S5), the phosphorus removal effect was 52% and 67%. This showed that the phosphorus removal effect was better after alkaline ferrate pretreatment. This is because, as the optimum pH for the formation of the Fe-P precipitate is 6 [36,37], the alkaline ferrate pretreatment group (S5) produced acid during digestion and the pH gradually decreased, reaching the optimum pH for the Fe-P precipitate. However, the acid ferrate pretreatment group (S4) needed additional alkali to adjust the pH, which would affect the phosphorus removal effect. In the process of digestion, the change in the TP concentration in the liquid phase was similar to that of PO_4_^3−^-P. The proportion of PO_4_^3−^-P exceeded 75%, which was beneficial for the formation of the Fe-P precipitate and the reduction of the phosphorus concentration. 

VFAs mainly come from the hydrolysis of organic matter during digestion. Different pretreatment methods had different effects on the VFA concentration (Figure 2). The changes in VFAs in the acid blank control group (S2) and the alkaline blank control group (S3) were similar to that in the blank group (S1). They all slowly increased within 5 days, which was a normal acid-producing reaction of digestion. In the first 4 days of digestion, VFAs pretreated with acid ferrate (S4) increased rapidly. When the highest concentration reached 304.82 mg COD/g VSS on the fourth day, 2.19 times that of the acid blank control group (S2) (138.85 mg COD/g VSS), then it began to decrease on the 5th day. After being pretreated with alkaline ferrate (S5), the maximum VFA accumulation (408.21 mg COD/g VSS) appeared on the third day, which was 3.08 times that of the alkaline blank control group (S3) (132.75 mg COD/g VSS). This meant that the addition of potassium ferrate promoted acid production. On the one hand, this may be because potassium ferrate has a strong oxidation performance. On the other hand, some scholars have found that iron can enrich Fe^3+^-reducing bacteria in the process of digestion and strengthen the transformation of complex organic matter into small-molecule acids and alcohols [38]. After alkaline ferrate pretreatment (S5), the maximum concentration of VFAs was 1.38 times that of acid ferrate pretreatment (S4), and the acid production rate was 1.39 times that of S4. It meant that alkaline ferrate pretreatment (S5) has a better acid production effect. In He et al.’s [15] experiment, after pretreatment with potassium ferrate and then digestion with fresh sludge, the maximum concentration of VFAs appeared on the fifth day, which was 343 mg COD/g VSS, while the maximum concentration of VFAs in this experiment appeared on the third day, which was 408.21 mg COD/g VSS. Compared with He et al. [15], this experiment obviously increased the accumulation of VFAs on the premise of shortening the hydrolysis and acidification time. From the fourth day, the concentration of VFAs after alkaline ferrate pretreatment (S5) decreased. This may be because the methanogenic bacteria began to use VFAs in the liquid phase to produce methane [39], which meant that S5 completed the hydrolysis and acidification process earlier than other groups. 

**Figure 3:**
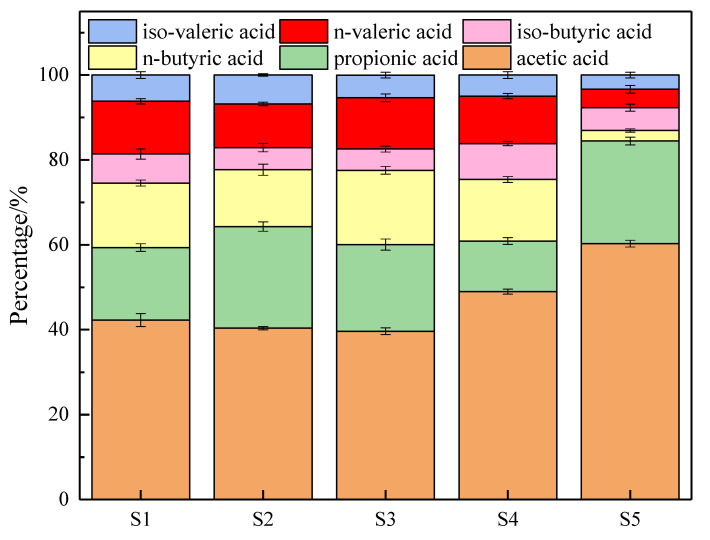
The proportion of six acids on the day of maximal VFA accumulation during digestion.

In the process of sludge anaerobic digestion, VFAs are mainly composed of acetic, propionic, n-butyric, iso-butyric, n-valeric, and iso-valeric acids [40]. The proportion of six acids in five groups on the third day (the day when the VFA accumulation reached the maximum) of digestion is shown in Figure 3. Acetic acid was the most widely used organic acid [41], and the percentage of acetic acid ranked first in all groups (42.26%, 40.36%, 39.65%, 49%, and 60.29%) because, in addition to acetic-acid-producing bacteria directly converting organic matter into acetic acid, other organic acids (propionic acid, butyric acid, valeric acid, etc.) can also be degraded into acetic acid under the catalysis of related enzymes. It can be seen that after pretreatment, the proportion of acetic acid in S4 and S5 was the largest. After pretreatment, the concentrations of propionic acid decreased after acidic ferrate pretreatment (S4) (11.89% vs. 23.94% in S2) and the concentrations of n-butyric acid decreased after alkaline ferrate pretreatment (S5) (2.49% vs. 17.47% in S3). After pretreatment, the total proportion of acetic acid and propionic acid in all groups increased (64.3%, 60.07%, 60.89%, 84.46% vs. 59.37% in S1). After 3 days of digestion, iso-butyric acid and iso-valeric acid changed the least. This may be because n-butyric acid and n-valeric acid are linear organic acids, while iso-butyric acid and iso-valeric acid have branched chains and find it more difficult to convert into other small molecular acids [42]. The data showed that ferrate pretreatment (S4, S5) not only accelerates acid production, increases VFAs concentration, and achieves the goal of efficiently accumulating small molecular acids, but also effectively increases the proportion of acetic acid. Compared with S4, S5 had a better acid production effect.

After 5 days of digestion, EPS all decreased (Figure 1c,d). EPS values of each group were 189.39 mg/L, 166.14 mg/L, 145.92 mg/L, 227.105 mg/L, and 213.37 mg/L, respectively. Compared with 5 days before, they had decreased by 41.75%, 64.75%, 71.06%, 80.92%, and 83.95%, respectively. Among them, most of the S-EPS and LB-EPS were degraded. As a necessary substance for acid production, the concentration of EPS decreased the most after alkaline ferrate pretreatment (S5), with a decrease of 83.95%, which also suggested that the acid production effect of this group was better.

#### 3.2.2. Solid Phase Analysis of Sludge during Digestion

After 5 days of digestion, the solid phase of the sludge changed (Table 4). Compared with the original sludge concentration, the VSS of the five groups decreased by 15.25%, 15.79%, 19.64%, 26.89%, and 29.45%, respectively, after 5 days of digestion. Compared with before digestion, SV_30_ in all five groups increased and SVI of all five groups increased too (from 83.60 mL/g to 139.3 mL/g, from 81.49 mL/g to 143.1 mL/g, from 77.87 mL/g to 165.2 mL/g, from 79.43 mL/g to 155.3 mL/g, and from 78.8 mL/g to 168.3 mL/g), which may be because the hydrolysis of the macromolecular solid into an insoluble macromolecular solid increased the volume of the solid phase. In addition, after pretreatment with ferrate (S4, S5), SVI increased the most, which may be because iron played a role in phosphorus removal during digestion, and flocculation declined. After 5 days of digestion, the Dx (50) of each group of data tended to be consistent. It can be seen that the unhydrolyzed and acidified parts of the crushed sludge gradually gather again during digestion, which may be due to the flocculation of iron and the secretion of viscous substances by microorganisms [42].

### 3.3. Microbial Characteristics Analysis

#### 3.3.1. Microbial Analysis at Phylum Level

**Figure 4:**
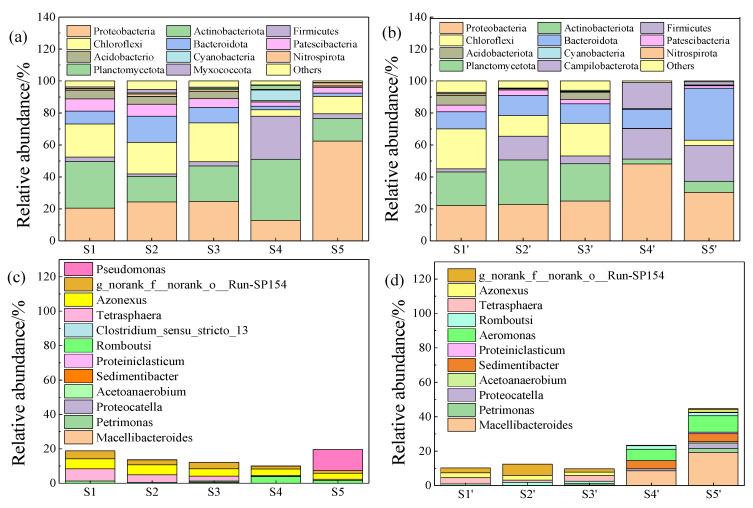
Effect of ferrate pretreatment and digestion on microbial proportion structure diagram ((**a**) phylum-level distribution of bacterial population after pretreatment, (**b**) phylum-level distribution of bacterial population after digestion, (**c**) genus-level distribution of bacterial population after pretreatment, (**d**) genus-level distribution of bacterial population after digestion).

The composition, diversity, and structure of bacterial communities after pretreatment and after 5 days of digestion were measured by high-throughput sequencing. S1, S2, S3, S4, and S5 are the microbial samples after pretreatment, and S1′, S2′, S3′, S4′, and S5′ are the microbial samples after 5 days of digestion (Figure 4). After different pretreatments, the composition of phylum microorganisms was different (Figure 4a). After pretreatment with ferrate (S4, S5), the sludge structure was destroyed and microorganisms were released. However, 39 identical categories were detected in all five groups, which indicated that this concentration of iron did not break the microbial cells and affected the microbial richness. Compared with the blank control group (S1), the microbial composition of the acid blank control group (S2) and the alkaline blank control group (S3) did not change much. Chloroflexi, Bacteroidota, Proteobacteria, Firmicutes, and Actinobacteriota changed greatly after pretreatment with ferrate (S4, S5). The total proportion of Chloroflexi and Bacteroidota decreased the most after alkaline ferrate pretreatment (S5) (12.87% vs. 34.02% in S3). The proportion of Proteobacteria changed differently after acid ferrate pretreatment (S4) and alkaline ferrate pretreatment (S5), as it decreased greatly for S4 (12.77% vs. 24.32% in S2) and increased sharply for S5 (62.34% vs. 24.59% in S3). The growth rate of Firmicutes after acidic ferrate pretreatment (S4) (27% vs. 1.53% in S2) was higher than that after alkaline ferrate pretreatment (S5) (2.91% vs. 2.66% in S3). Actinobacteriota did not change after alkaline ferrate pretreatment (S5) but increased a great deal after acidic ferrate pretreatment (S4) (38.17% vs. 16.01% in S2).

After 5 days of digestion, the microbial composition at the phylum level changed (Figure 4b), especially Actinobacteriota, Proteobacteria, Firmicutes, Chloroflexi, and Bacteroidota. Microorganisms in Firmicutes such as protease and cellulase can secrete extracellular hydrolases to accelerate cell hydrolysis [43]. The proportion of Actinobacteriota in the three blank control groups all exceeded 20% after digestion (20.94%, 27.77%, and 23.3%), but rapidly decreased after pretreatment with ferrate (3%, 6.94%), which indicated that iron may inhibit the growth of Actinobacteriota. The data showed that after 5 days of digestion, the total proportion of the other four main microbial species (Proteobacteria, Firmicutes, Chloroflexi, and Bacteroidota) in the blank control group (S1′) increased from 51.86% to 59.81%, increased from 46.02% to 79.26% after acid ferrate pretreatment (S4′), and increased from 78.12% to 88.5% after alkaline ferrate pretreatment (S5′). It was found that Proteobacteria, Firmicutes, Chloroflexi, and Bacteroidota are the main microorganisms in traditional anaerobic digestion [44], which can decompose macromolecular organic matter and promote VFA synthesis [45]. After pretreatment with ferrate, the proportion of hydrolytic microorganisms increased. In the process of digestion, the five groups of the main hydrolytic microorganisms all increased, which may be because other microorganisms did not adapt to the environment and died. Hydrolytic microorganisms in three blank control groups grew slowly. On the one hand, it may be because they are wrapped in the sludge structure, and on the other hand, it may be because they do not have enough nutrients that are easy to take in. Hydrolytic microorganisms after ferrate pretreatment (S4, S5) increased rapidly, mainly because ferrate destroyed the structure of sludge, making it easier to use macromolecular organic matter as a small-molecule substance and promoting the growth of digestive microorganisms.

#### 3.3.2. Microbial Analysis at Genus Level

After pretreatment, compared with the blank control group (S1) (7.02%), *Tetrasphaera* decreased (4.38%, 2.86%, 0.5%, and 0.72%) while the other microorganisms barely changed (Figure 4c). 

After 5 days of digestion, the proportion of representative acid-producing bacteria at the genus level in the three blank groups (S1′, S2′, and S3′) increased slightly, but after pretreatment with ferrate (S4′, S5′) it increased a great deal (Figure 4d). *Proteocatella*, *Sedimentibacter*, *Macellibacteroides*, and *Petrimonas* are the main hydrolysis-acidification functional microorganisms at the genus level. *Proteocatella* and *Sedimentibacter* under Firmicutes are protein-decomposing bacteria and sedimentation bacteria, respectively. They are the main flora of acetic-acid-producing bacteria, which can hydrolyze protein and starch into small molecular acetic acids [46]. *Macellibacteroides* and *Petrimonas*, which belong to Bacteroidetes, are typical acid-producing bacteria. They can hydrolyze macromolecules into VFAs such as acetic acid and butyric acid [47]. After 5 days of digestion, three blank control groups (S1′, S2′, and S3′) had little change in the total proportion of these four bacteria. However, for S4′, it changed a great deal (the proportion of *Proteocatella* increased from 0 to 1.19%, *Sedimentibacter* increased from 0 to 4.68%, *Macellibacteroides* increased from 0 to 8.63%, and *Petrimonas* did not change much). For S5′, after 5 days of digestion, representative acid-producing bacteria increased (the proportion of *Proteocatella* increased from 0 to 3.09%, *Sedimentibacter* increased from 0 to 4.71%, *Macellibacteroides* increased from 0.01% to 19.16%, and *Petrimonas* increased from 0 to 2.49%). After the oxidation of ferrate, a great deal of organic matter was released, and the available nutrients of microorganisms were increased, following which the growth rate was gradually accelerated, which may be the main reason for the increase in hydrolytic microorganisms. Furthermore, the microorganism belonging to *Azonexus* is mainly Betaproteobacteria [48], which is the main consumer of VFAs, and it can convert VFAs into methane and other substances. After 5 days of digestion, all five groups of *Azonexus* showed a decline (from 5.87% to 2.78%, from 5.79% to 2.74%, from 4.28% to 2.05%, from 1.79% to 0.15%, and from 3.38% to 1.52%), especially pretreatment with ferrate (S4′, S5′). The decline of *Azonexus* for S4′ and S5′ confirmed that this concentration of iron could inhibit the consumption of VFAs and indirectly increase the accumulation of VFAs.

Therefore, from the change in the microbial community, an appropriate amount of potassium ferrate will not break the sludge cells and affect the microbial activity during digestion. It simply enhances the community function by changing the microbial community structure, reducing the biodiversity in the digestion process, and producing specific acid-producing bacteria. This could directly accelerate the hydrolysis of organic matter and increase the maximal accumulation of VFAs when the substrate is sufficient.

#### 3.3.3. Correlation Analysis of Environmental Factors and Microorganisms

**Figure 5:**
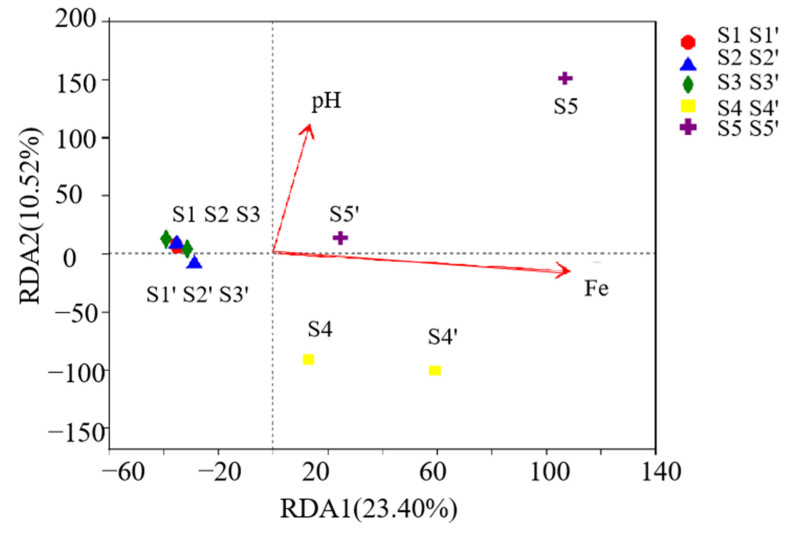
Correlation analysis of acid-producing microorganisms (RDA: Redundancy analysis).

There was a correlation between microorganisms at the genus level and different environmental factors (Figure 5). The influencing factors are pH and adding Fe. R^2^ related to pH is 0.9997 and that related to the addition of Fe is 0.9994. After pretreatment and digestion for 5 days, the microorganisms of three blank control groups (S1 and S1′, S2 and S2′, S3 and S3′) changed slightly, while those of ferrate pretreatment (S4 and S4′, S5 and S5′) changed a great deal, and the main influencing factor was iron. The results showed that the addition of Fe was the main influencing factor, which had a correlation with microbial changes. Iron-added components all promoted the growth of microorganisms, and the influence of alkaline ferrate pretreatment (S5′) was greater than acid ferrate pretreatment (S4′). For alkaline ferrate pretreatment (S5 and S5′), pH had a positive correlation, while for acid ferrate pretreatment (S4), pH had a negative correlation, which showed that, in this experiment, the effect of acid ferrate pretreatment (S4 and S4′) on promoting microbial growth was not as good as alkaline ferrate pretreatment (S5 and S5′). At the same time, from the three blank groups, pH had only a slight effect on them.

## 4. Conclusions

After acid and alkaline ferrate pretreatment, the particle size of the sludge became smaller and EPS was destroyed and released into the liquid phase. After alkaline ferrate pretreatment, compared with the blank control groups, the VSS decreased the most (28.19%) and the VFAs in the sludge liquid phase reached 408.21 mg COD/g VSS within only 3 days, which was 5.34 and 3.08 times higher than the maximum value of the blank control group and the alkaline blank control, respectively. At the same time, considering *Proteocatella*, *Sedimentibacter*, *Macellibacteroides*, and *Petrimonasas* hydrolytic acidification bacteria, the abundance was greatly improved.

## Figures and Tables

**Table 1:** Concentration and supernatant characteristics of the excess sludge.

Parameters	Value (mg/L)	Parameters	Value (mg/L)
SS	10000 ± 5	NH_4_^+^-N	0.4 ± 0.1
VSS	6100 ± 2	TN	6.86 ± 0.5
COD	62 ± 1	PO_4_^3−^-P	21.54 ± 0.5
TOC	16.51 ± 0.5	TP	21.94 ± 1

**Table 2:** Changes in several basic parameters in the liquid phase after pretreatment (unit: mg/L).

Samples	S1	S2	S3	S4	S5
NH_4_^+^-N	2 ± 0.5	2 ± 0.5	10 ± 1	32 ± 1	45 ± 1
TN	21 ± 0.5	19 ± 0.5	41 ± 0.5	61 ± 1	82 ± 2
PO_4_^3−^-P	12 ± 0.5	14 ± 0.5	24 ± 0.5	64 ± 1	84 ± 2
TP	14 ± 0.5	18 ± 0.5	30 ± 1	84 ± 1	104 ± 2
COD	62 ± 2	71 ± 1	500 ± 4	1450 ± 5	2700 ± 5

**Table 3:** Solid-phase changes of excess sludge after pretreatment.

Parameters	S1	S2	S3	S4	S5
VSS (g/L)	6.10 ± 0.5	5.89 ± 0.5	5.65 ± 0.5	5.02 ± 0.5	4.38 ± 0.5
Dx (50) (μm)	1749 ± 5	1674 ± 2	1605 ± 2	69.03 ± 1	46.87 ± 1
SV_30_ ratio (%)	51 ± 1	48 ± 1	44 ± 1	34 ± 1	29 ± 1
SVI (mL/g)	83.60 ± 1	81.49 ± 1	77.87 ± 1	79.43 ± 1	78.80 ± 1

**Table 4:** Solid-phase changes of excess sludge after digestion.

Parameters	S1′	S2′	S3′	S4′	S5′
VSS (g/L)	5.17 ± 0.2	4.96 ± 0.2	4.54 ± 0.2	3.67 ± 0.2	3.09 ± 0.2
Dx (50) (μm)	1497 ± 5	1596 ± 5	1578 ± 4	1674 ± 5	1421 ± 5
SV30 ratio (%)	72 ± 1	71 ± 1	75 ± 1	57 ± 1	54 ± 1
SVI (mL/g)	139.3 ± 1	143.1 ± 1	165.2 ± 2	155. 3 ± 1	168.3 ± 1
